# Optimization of ultrastructural preservation of human brain for transmission electron microscopy after long post-mortem intervals

**DOI:** 10.1186/s40478-019-0794-3

**Published:** 2019-09-03

**Authors:** Mariella Sele, Stefan Wernitznig, Saška Lipovšek, Snježana Radulović, Johannes Haybaeck, Anna Maria Birkl-Toeglhofer, Christina Wodlej, Florian Kleinegger, Stephan Sygulla, Marlene Leoni, Stefan Ropele, Gerd Leitinger

**Affiliations:** 1 Research Unit Electron Microscopic Techniques, Division of Cell Biology, Histology and Embryology, Gottfried Schatz Research Center, Medical University of Graz, Neue Stiftigtalstrasse 6, 8010 Graz, Austria; 2 Department of Biology, Faculty of Natural Sciences and Mathematics, University of Maribor, Koroška 160, 2000 Maribor, Slovenia; 3 Faculty of Medicine, University of Maribor, Taborska ulica 8, 2000 Maribor, Slovenia; 4 Faculty of Chemistry and Chemical Engineering, University of Maribor, Smetanova ulica 17, 2000 Maribor, Slovenia; 5 Division of Molecular Biology and Biochemistry, Gottfried Schatz Research Center, Medical University of Graz, Neue Stiftigtalstrasse 6, 8010 Graz, Austria; 6 Diagnostic and Research Institute of Pathology, Diagnostic & Research Center for Molecular BioMedicine, Medical University of Graz, Neue Stiftigtalstrasse 6, 8010 Graz, Austria; 7 Department of Pathology, Medical Faculty, University of Magdeburg, Leipziger Straße 44, 39120 Magdeburg, Germany; 8 Institute of Pathology, Neuropathology and Molecular Pathology, Medical University of Innsbruck, Müllerstraße 44, 6020 Innsbruck, Austria; 9 Department of Neurology, Medical University of Graz, Auenbruggerplatz 22, 8036 Graz, Austria

**Keywords:** Electron microscopy, High pressure freezing, Freeze substitution, Post-mortem interval, Human brain

## Abstract

Electron microscopy (EM) provides the necessary resolution to visualize the finer structures of nervous tissue morphology, which is important to understand healthy and pathological conditions in the brain. However, for the interpretation of the micrographs the tissue preservation is crucial. The quality of the tissue structure is mostly influenced by the post mortem interval (PMI), the time of death until the preservation of the tissue. Therefore, the aim of this study was to optimize the preparation-procedure for the human frontal lobe to preserve the ultrastructure as well as possible despite the long PMIs. Combining chemical pre- and post-fixation with cryo-fixation and cryo-substitution (“hybrid freezing”), it was possible to improve the preservation of the neuronal profiles of human brain samples compared to the “standard” epoxy resin embedding method. In conclusion short PMIs are generally desirable but up to a PMI of 16 h the ultrastructure can be preserved on an acceptable level with a high contrast using the “hybrid freezing” protocol described here.

## Introduction

The resolving power of the transmission electron microscope (EM) is necessary for many functional studies of the nervous system. Transmission EM provides not only the ability to study the anatomical strength of synapses, the arrangement of the synaptic vesicles, but also enables us to study the fine structural distribution of proteins, or the analysis of the chemical elements within the tissue [19]. In human brains, it allows for the detection of possible structural alterations in organelles or within the neuronal profiles which may contribute to many brain disorders [6, 27, 41]. For example in multiple sclerosis, the immune system destroys the myelin, and in animal models, the myelin is regenerated by newly generated oligodendrocytes, but the remaining mature oligodendrocytes do not seem to contribute to this process [38]. However, due to the huge differences between humans and rodents especially in the dynamics of oligodendrocyte and myelination, investigations of human samples are crucial for understanding these dynamics. For such studies, excellent tissue preservation is essential in order to be able to interpret the findings correctly. This is especially challenging in human post-mortem brain studies where there is often a considerable delay between the time of death of the patient and the fixation of the tissue samples. During this so-called post mortem interval (PMI), the ultrastructure of the brain tissue is degraded by autolytic processes. Furthermore, the morphology of organelles, e. g. mitochondria, not only indicates the condition of the tissue, but can also be a consequence of the progression of neurodegenerative diseases [28]. Therefore, the longer the PMI, the more difficult it will be to correlate morphological changes with specific events. In human samples, for ethical reasons, the PMI can range from a few hours up to 100 h [10, 13, 16, 39], during which the degradation of the tissue continues until fixation. The PMI is one of the key factors for the preservation of the neuronal profiles [7]. However, interestingly, longer PMIs do not have an effect on the proportion of myelin proteins [1]. The standard approach for EM sample preparation is done with chemical fixation, using aldehydes, followed by post fixation with osmium tetroxide and dehydration often at room temperature or at 4 °C, followed by embedding in epoxy resins. An alternative to the standard preparation approach is rapid cryo-fixation (high pressure freezing, HPF) in combination with freeze substitution (FS). During high pressure freezing, 2100 bar and liquid nitrogen are applied to the sample, which becomes completely vitrified while suppressing the formation of ice crystals. During freeze substitution, the vitrified water in the sample is slowly substituted with organic solvents at low temperature. A great advantage is that HPF achieves an almost native ultrastructural preservation [35] of unfixed tissue due to the high freezing rate of the tissue [23, 33] that helps to avoid the formation of non-vitreous ice crystals. In various animal models, it was shown that the structural preservation of the myelin with high-pressure freezing is excellent [26] compared to aldehyde perfusion fixation [15, 25]. The dehydration of the samples is a highly critical step regarding the preservation of the ultrastructure of the tissues. Freeze substitution has been described to be the least harmful dehydration approach [24] due to a very low starting temperature and the very slow (hours or days) warming up period of the samples. It is assumed that the hydration shell of the proteins is partially preserved, and therefore the dehydration process is as gentle as possible. The efficiency of the dehydration or more specifically the freeze substitution can be increased with continuous agitation [8, 9, 29]. This often lengthy protocol can be shortened in time [32] and shows high contrast especially in difficult samples. Over time, various combinations of HPF and FS protocols have been described, each of them suitable for a particular research question and/or a specific sample material. Tsang et al. [34] adapted HPF and FS to use the samples with fluorescence imaging and en-bloc contrast. Other authors combine them with immunogold labeling [11, 24] by rehydrating the samples in the process before the antibody incubations. Sosinsky et al. [32] reported an improvement of the ultrastructure of neurons, injected with photooxidized Lucifer Yellow and fixed with a combination of glutaraldehyde and HPF. We searched for a standard protocol for EM preparation of human post mortem brain samples. Having tested several different approaches thoroughly and systematically, we established a novel protocol that includes chemical fixation of the samples, post fixation using osmium tetroxide, high pressure freezing, and freeze substitution in a cocktail of osmium tetroxide and uranyl acetate dissolved in acetone.

## Materials and methods

### Tissue preparation

Small samples were provided by the Institute of Pathology of the Medical University of Graz and taken from the frontal lobe of four human post-mortem brains. Inclusion criteria were as follows: (i) the patients had not suffered from any known neurodegenerative disorders; and (ii) the samples showed no macroscopically detectable lesions. Staining was carried out following the protocol of Braak et al. [2]. The samples used are shown in Table 1, indicating the post-mortem interval PMI, from time of death to fixation and Braak staging of the tissue. For all fixation and embedding variants, the brain tissue was cut into pieces of approximately 1 cm^3^ for vibratome sectioning, followed by fixation in 2% paraformaldehyde and 2.5% glutaraldehyde in 0.1 M dimethyl arsenic acid sodium buffer (cacodylate buffer) pH 7.4 for 24 h with one exchange of the fixative solution. The pre-fixed samples were cut with a vibratome (Leica, Vienna, Austria) into 150 μm sections, and the regions of interest (ROIs) were cut out with a 4 mm biopsy punch. All the ROIs were taken from the frontal human cortex, case 1 from the grey matter layer 6, cases 2–4 randomly chosen from frontal white matter from underneath a single gyrus each. After punching out, the tissue samples were post fixed with 2% osmium tetroxide (Electron Microscopy Sciences, Hartfield, USA) in 0.1 M cacodylate buffer pH 7.4 for 15 min.

**Table 1:** Overview of the human post-mortem brain samples

Comment	Age	Sex	PMI [h]	Braak staging
Case 1	61	Female	16	0
Case 2	69	Female	18	0
Case 3	70	Male	20	5
Case 4	81	Male	24	6
Case 5	74	Male	9	0

### Standard chemical fixation and resin embedding

The osmium-treated sections were dehydrated in a series (50–96%) of graded ethanol for 20 min each. Afterwards, the samples were exposed to 100% ethanol for 10 min and propylene oxide for 40 min. The resin infiltration started with 50% TAAB embedding resin (TAAB Laboratories Equipment Ltd., UK) in propylene oxide for 2 h, followed by 66% TAAB embedding resin in propylene oxide over night at 4 °C and finally two changes of pure TAAB embedding resin at 45 °C for 1 h. The sections were embedded in silicone forms and polymerized at 60 °C for 72 h (Table 2). Semi-thin sections of 500 nm were acquired and stained with a 1% toluidine blue solution (Sigma-Aldrich, USA) to confirm the area of interest under the light microscope (Olympus BX63) for trimming. Ultra-thin sections were cut on an ultramicrotome (Leica, Vienna, Austria) at 70 nm and collected on 300 mesh copper grids. The grids were stained with lead citrate and platinum blue prior to EM imaging (Ultrastainer, Leica). After ultra-thin sectioning, a 500 nm thick section of the trimmed area was cut, stained with toluidine blue (Sigma-Aldrich) and cover slipped, to analyze the quality of the sample under the light microscope.

### Cryo-processing and cryo-substitution

The samples which had been punched out of the vibratome sections were high-pressure frozen with an HPM100 high pressure freezer (Leica) sandwiched in 6 mm aluminum carrier suitable for high pressure freezing (Engineering Office M. Wohlwend, Sennwald, Switzerland) with 1-hexadecene (Sigma-Aldrich) as a filler to avoid air bubble formation. The freeze substitution was done with an AFS2 (Leica, Vienna, Austria) and with the usage of an agitation device [9]. Three different substitution cocktails were used. Cocktail I contained 2% osmium tetroxide and 0.2% uranyl acetate in water free acetone; cocktail II contained 0.2% uranyl acetate in water free acetone, and cocktail III contained pure acetone only, as shown in Table 3. The agitation device was used with 12 V, and ethanol was used as a mediator medium. The frozen samples were incubated for 8 h at − 90 °C in the substitution cocktail within the freeze substitution. The samples were then gradually warmed up to − 60 °C with an increase of 30 C per hour and maintained at this temperature for 8 h. Afterwards, the temperature of the samples was gradually raised to − 30 °C with an increase of 30 °C per hour and maintained for 8 h. The heating of the samples from − 30 °C to 0 °C took 1.5 h. The samples were washed three times in acetone at room temperature and then placed into 50% TAAB embedding resin in acetone for 2 h, followed by 66% TAAB embedding resin overnight. After two rounds of incubation in pure TAAB embedding resin for 2 h at 45 °C each, the samples were polymerized at 60 °C for 3 days.

**Table 2:** Post-mortem brain tissue processing for electron microscopy

Step	Standard fixation and resin embedding	Hybrid freezing method
1	Prefixed with 2% paraformaldehyde + 2.5% glutaraldehyde in 0.1 M cacodylate buffer for 24 h at 4 °C	
2	Vibratome sections at 150 μm, ROI punched out	
3	Post-fixation with OsO_4_ 0.1 M cacodylate buffer for 15 min at RT	
4	Washing in 0.1 M cacodylate buffer	High pressure freezing with hexadecane as cryoprotectant
5	Dehydration in ascending ethanol series	Freeze substitution over 27 h in different substitution cocktails
6	Intermedium propylene oxide	Washing in acetone
7	Resin infiltration with ascending resin series in propylene oxide	Resin infiltration with ascending resin series in acetone
8	Resin polymerization at 60 °C for 72 h in oven	
9	Ultra-thin sectioning at 70 nm and stain grids with lead citrate and platinum blue	
10	Electron microscopic examination of ultra-thin sections

**Table 3:** Overview of the different cryo-substitution cocktails

	Cryo-substitution media
Cocktail I	2% osmium tetroxide and 0.2% uranyl acetate in water free acetone
Cocktail II	0.2% uranyl acetate in water free acetone
Cocktail III	water free acetone

### Electron microscopic image acquisition

To reduce observer bias, all the samples were double blinded and randomized (https://www.randomizer.org/) for microscopy and evaluation. The evaluation of the samples was done by four different experts. The sections were examined with an FEI Tecnai G2 20 (Thermo Fischer Scientific, USA) at 120 kV. Two consecutive sections with a thickness of 70 nm were examined for each sample. Serial EM software was used to create a map of both sections. Three points within the maps were randomly selected. To obtain a better overview of the general amount of lysis in the samples, a montage with nine images (3 × 3) at magnification X1500 was created using Serial EM Software and a 2 K × 2 K CCD camera (Ultrascan 1000, Gatan, Pleasanton, USA) at each of these three points. To further analyze the ultrastructure and to determine the g-ratios and the quality of the preservation of the myelin sheaths, three micrographs with a magnification of X6500 were captured with the same camera using DigitalMicrograph™ software (Gatan, USA), amounting to a total of 18 micrographs at X6500 magnification for each of the 16 specimens. The brightness and the contrast of the images were adjusted with Adobe Photoshop (Adobe Systems, MountainView, USA).

### Electron microscopic analysis of g-ratios

The ratio between the outer diameter of the myelin sheath and the axon diameter (g-ratio) was analyzed with the GRatio software. GRatio is a plug-in for ImageJ and allows semi-automated analysis. The software is available online (http://gratio.efil.de, https://imagej.nih.gov/ij). From 10 randomly selected axons per sample in the 3 × 3 montages, the inner and outer diameters of the myelin sheaths were measured. Using ImageJ a pointed grid with 1024 points and random offset was projected onto the micrographs (6500X magnification). Each point which fell on a myelinated axon was counted and assigned to one of three classes. The first class (“intact”) contained points which located on well-preserved parts of the myelin sheaths, cut at cross section, with no gaps between the layers. The second class (“disbanded”) contained points that fell into disbanded myelin sheaths, with increased spacing between the layers and separated clusters of proteins. The third class (“unspecified”) contained parts of the myelin sheaths that could not be assigned to class 1 or class 2 due to the space between the layers could not be assessed, either because the sheaths were not cut in cross section or because the contrast was lacking, see Fig. 3.

### Analysis of axon density

The number of myelinated axons visible in the images was counted in the overview montages in nine single images (3 × 3) at magnification X1500, image size 129.5 μm^2^. Per sample five (standard embedding, case 4; cocktail I, case 2) or six images (remaining samples) were used for counting myelinated axons. The axon density was calculated as the number of axons per square micrometer.

### Statistical analysis

All statistical analyses were done in IBM® SPSS® Statistics (version 25) and GraphPad Prism (version 8.0.2). In order to find statistically differences between the brain samples with the different preparation methods, a repeated measures one-way ANOVA was followed by a post-hoc analysis (Scheffé) using a significance level of *p* = 0.05. Data are shown in box plots with Tukey whiskers.

## Results

### The hybrid freezing method

To achieve an adequate ultrastructural preservation, we developed a combined protocol of chemical fixation, post-fixation, cryofixation, and cryo-substitution, which we refer to as hybrid freezing method (HFM). The hybrid freezing method contained aldehyde fixation, post-fixation with osmium tetroxide, high pressure freezing (HPF), and cryo-substitution with three different cryo-substitution cocktails, and was compared to the standard method, an aldehyde fixation followed by dehydration at room temperature.

**Fig. 1:**
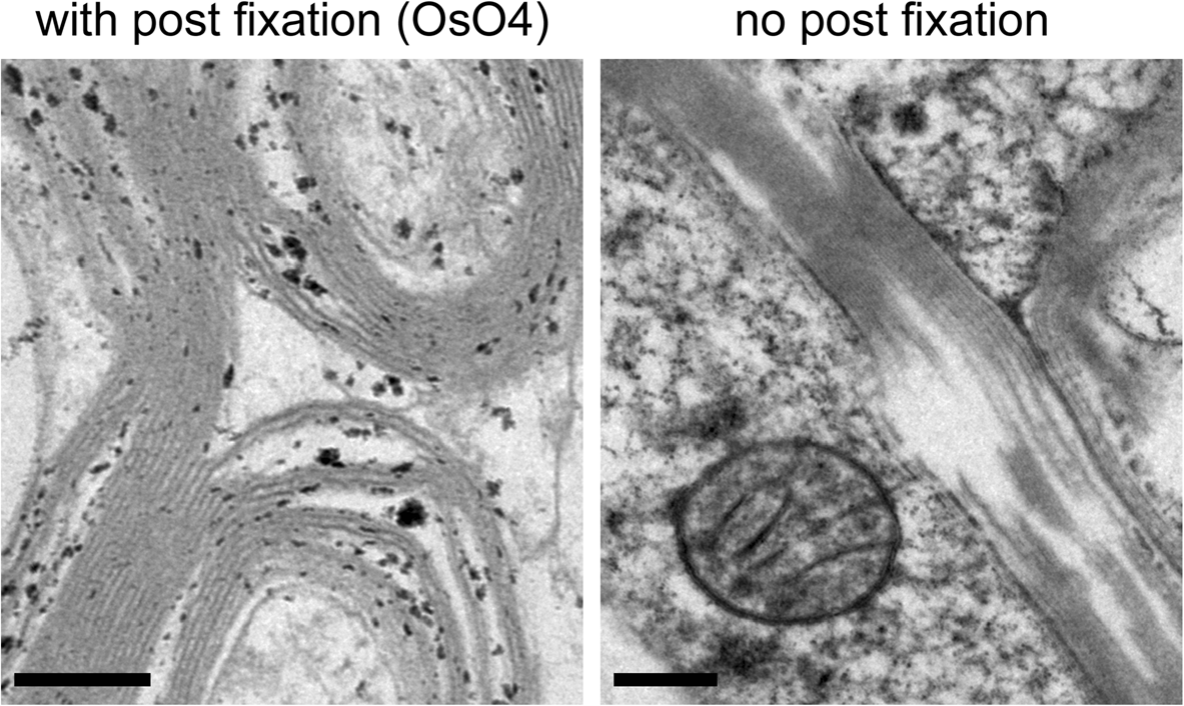
Electron micrographs of human brain samples (case 5) with and without post fixation with osmium tetroxide before the HPF. On the left side, the layers of the myelin sheath remain compact compared to the sample without post-fixation on the right side. In the left image, some proteins with good accessibility showed strong staining. Both samples were prepared with the cryo-substitution cocktail I; Scale bars = 0.2 μm

A first test comparing a sample that had been post fixed in osmium tetroxide prior to freezing with another sample without post fixation showed that post-fixation with osmium tetroxide is an important step towards the preservation of the myelinated axon in human brain tissue (Fig. 1). In the left panel of this figure, the individual layers of the myelin sheath can be recognized, and they remain compact compared to the sample that had not been post fixed (shown in the right panel).

### Comparison of standard processing with hybrid freezing method (HFM) using three different cryo-substitution cocktails

**Fig. 2:**
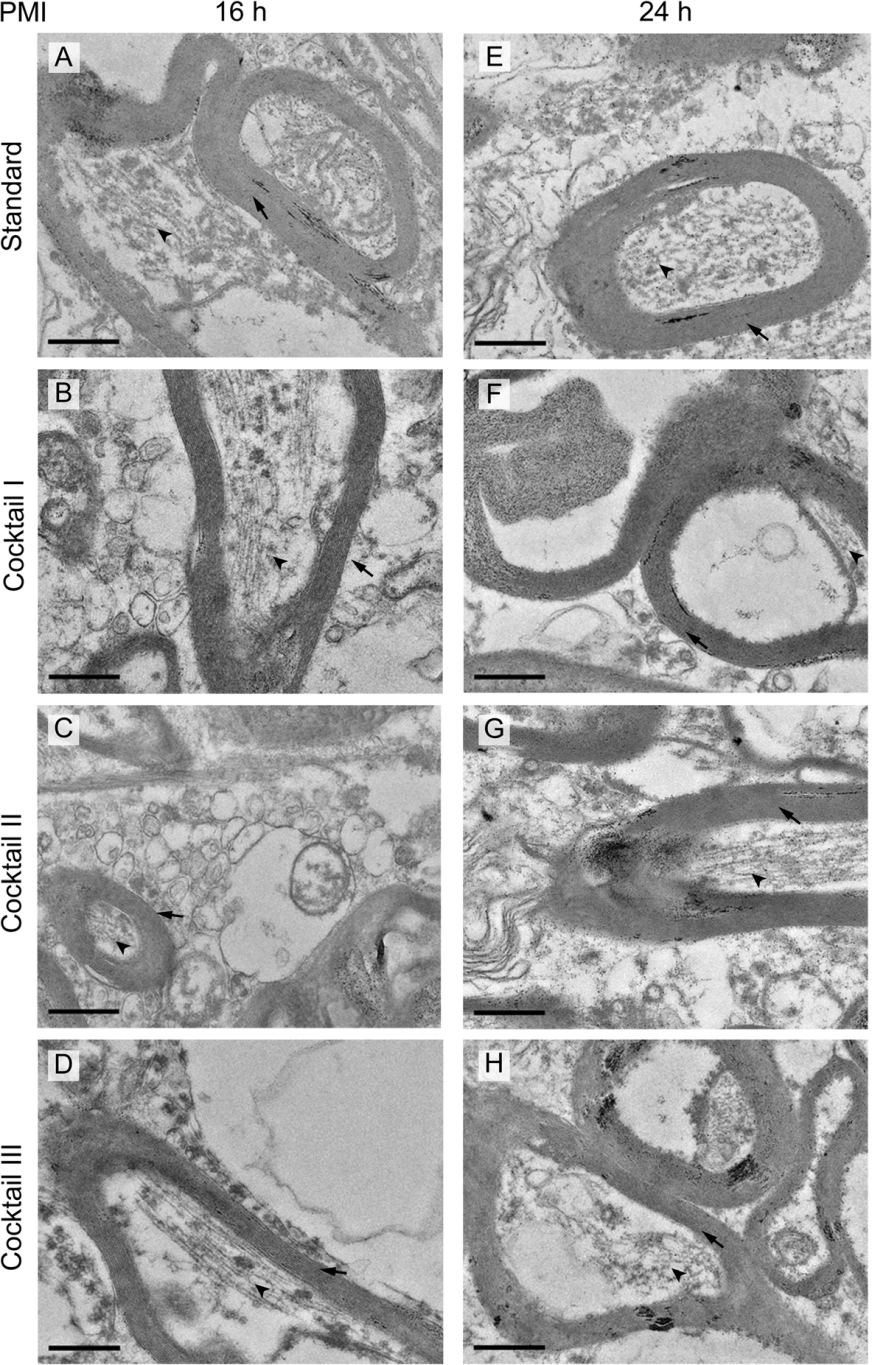
Electron micrographs of human brain samples with different PMIs and cryo-substitution cocktails at X6500 magnification. **a**-**d** Frontal lobe from case 1 (PMI 16 h), **a** prepared with standard embedding method shows two myelinated axons; **b** After hybrid freezing with freeze substitution one, the myelinated axons have tightly wrapped myelin sheaths (arrows) with clearly visible microtubules inside the axons (arrow heads) and a strong overall contrast. **c** and **d** Samples prepared with substitution cocktail II (**c**) and cocktail III (**d**), exhibiting lower contrast and less preserved myelin sheath than (**b**). **e**-**h** Frontal lobe samples from case 4 (PMI 24 h), **e** prepared by standard embedding, **f** processed with cryo-substitution cocktail I (hybrid freezing), **g** hybrid freezing with cocktail II, and (**h**), hybrid freezing with cocktail III. **f** Shows the strongest contrast, but the myelin sheaths exhibit only a slight increase in preservation between (**e**) and (**f**) and no clear change in preservation in samples (**g**) and (**h**). Scale bars = 0.5 μm

At first, we examined the general ultrastructural preservation of frontal lobe samples taken from four patients with difference in PMIs. It showed that the myelin sheaths remained visible regardless of the post-mortem times, and thus the myelin sheaths appeared more resistant against autolysis (compare Fig. 2a-d with Fig. 2e-h) than other cell components. Therefore, we focused on the myelin sheaths during our detailed comparison of four different embedding procedures for brain samples. A detailed analysis was performed by two independent researchers in a double-blinded approach. This analysis showed that the samples prepared with substitution cocktail I, which contains osmium tetroxide and uranyl acetate, showed the highest contrast in each sample (compare Fig. 2b and f with all the other panels of this figure).

### Preservation of myelin sheaths in the human frontal lobe

**Fig. 3:**
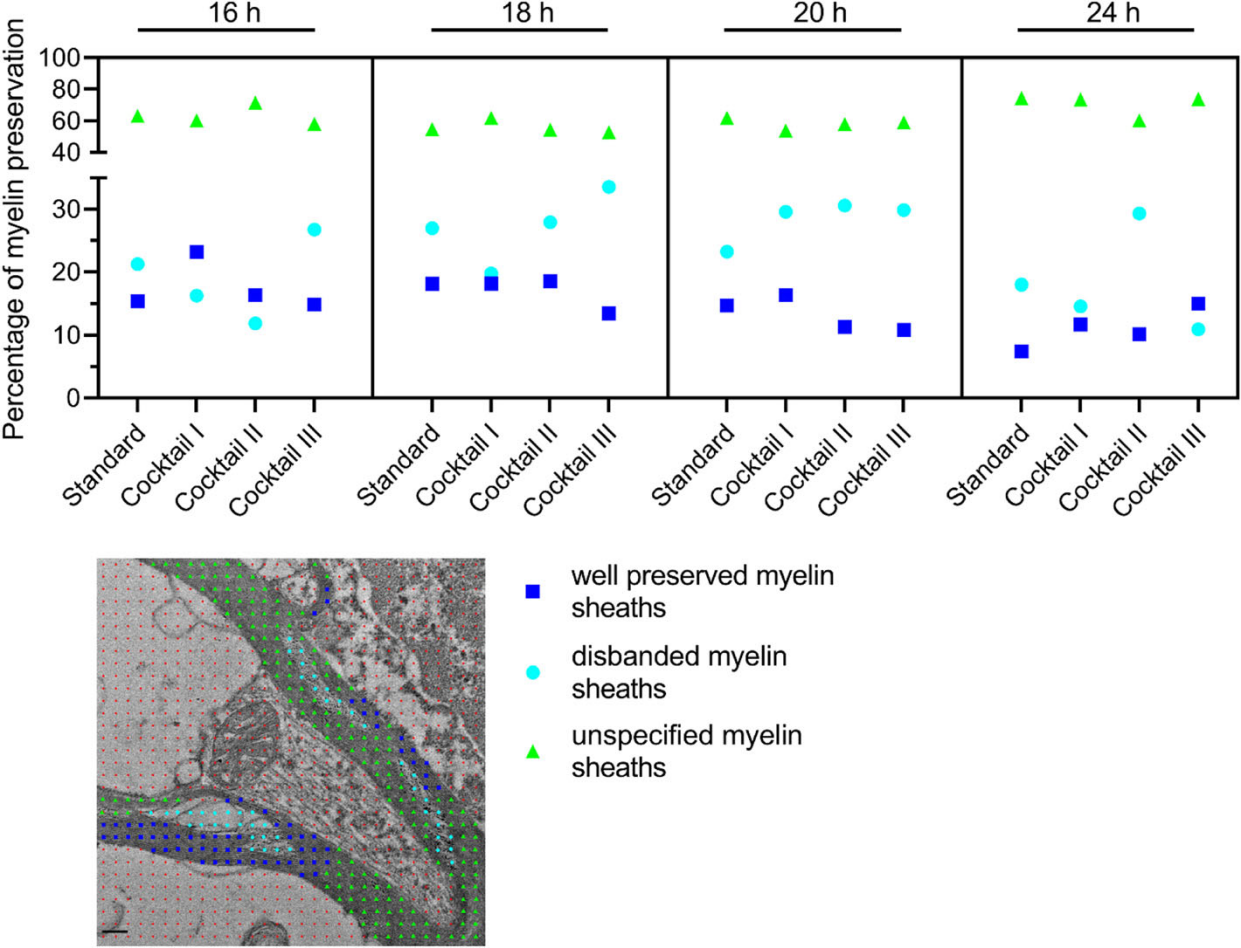
Quantification of the quality of the preserved myelin sheaths with different substitution cocktails compared to standard embedding. Analysis of the quality of the myelin sheath preservation, well-preserved myelin sheaths in blue squares, disbanded myelin sheaths are cyan dots, and unspecified myelin sheaths are shown in green triangles. Representative EM image with a red point grid and the color-marked distinction of the myelin sheaths, scale bar 0.2 μm

Focusing on the myelin sheaths, we tested if the novel hybrid freezing method (HFM) preserves the myelin sheaths better than standard embedding. An assessment of myelin sheath preservation was done with a point counting method [12], estimating the percentage of the myelin sheath volume that appeared well preserved, disbanded, or unspecified in each brain sample with each of the four processing procedures. Figure 3 shows that the PMI appeared to have an influence on the preservation of the myelin sheaths: the shorter the PMI, the higher the percentage of well-preserved parts of the myelin sheaths. Moreover, in three out of four cases, the HFM with cryo-substitution cocktail I showed a clear increase in well-preserved myelin sheath volume with respect to classical embedding (Fig. 3). This increase was largest in the sample with the lowest post-mortem time (case 1, from 15.4% in standard preparation to 23.2% with cocktail I). In case 2, cocktail 1 apparently neither increased nor decreased the percentage of well-preserved myelin sheaths with respect to classical embedding (18.2% of the myelin sheath volume appeared well preserved, both after standard embedding and after using substitution cocktail I, Fig. 3, second panel). Case 3 with a PMI of 20 h and case 4 (PMI of 24 h) showed a lower incidence of well-preserved myelin sheaths and a lower increase of well-preserved myelin sheaths when applying cocktail I (from 14.7 to 16.4% and from 7.4 to 11.7%, respectively, Fig. 3 right side). Conversely, the percentage of disbanded myelin sheaths decreased from 21.3% in the standard embedded sample to 16.4% in the sample processed with cocktail I. An exception was Case 2, in which the application of cocktail I apparently did not change the myelin sheath preservation (18.2% both after standard embedding and after using substitution cocktail I, Fig. 3, second panel). In contrast to cocktail I, no trend was visible when determining the percentage of well-preserved myelin sheaths after freeze substitution in cocktails II and III, both solutions either increased or decreased the percentage of well-preserved myelin sheaths (Fig. 3).

### Influence of post-mortem interval on ultrastructure of human brain tissue

**Fig. 4:**
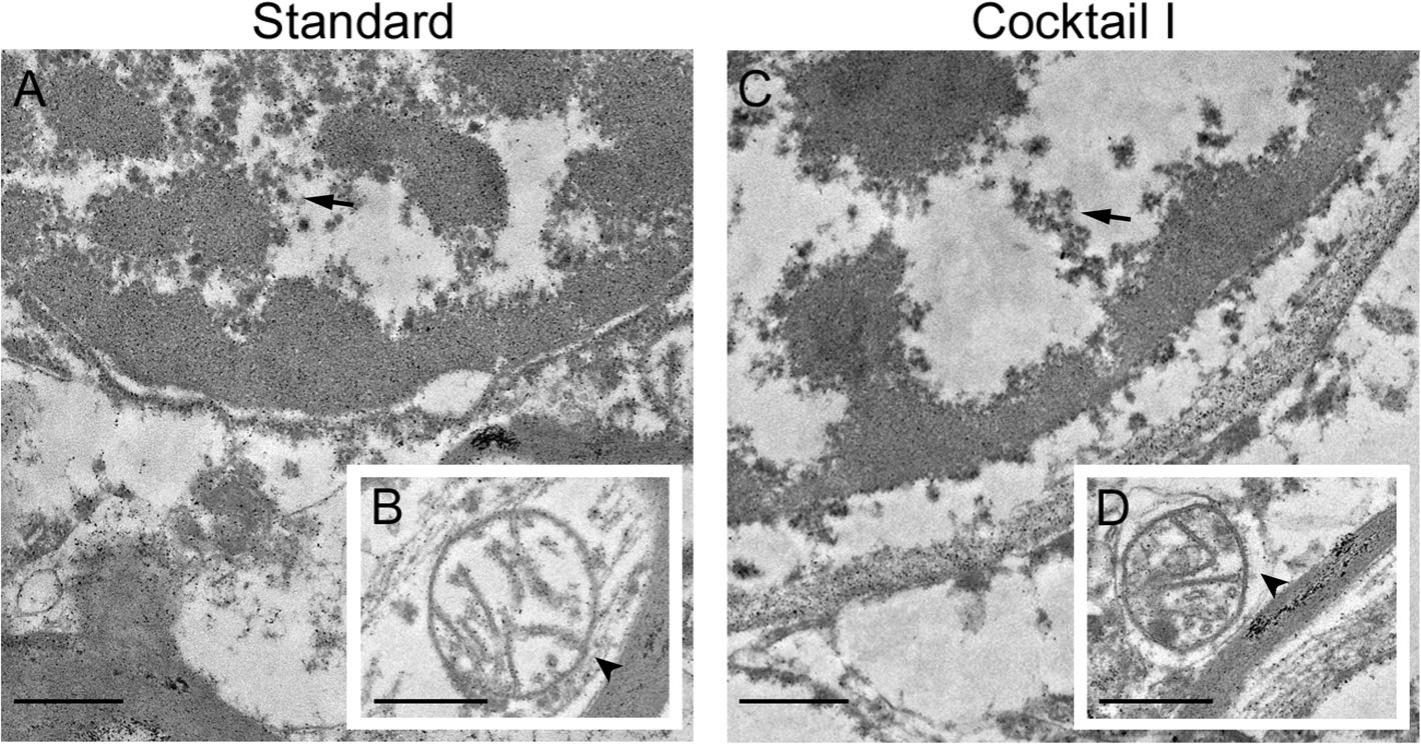
Ultrastructural examples of autolysis of human brain samples with long PMI prepared with standard embedding and hybrid freezing. Tissue sample from case 4, where the chromatin within the nuclei turns coarse (visible in **a** and **c**, arrows), and the mitochondria swell (visible in **c** and **d**, arrow heads). Both phenomena were visible either with standard embedding (**a**, **b**), or with hybrid freezing and cryo-substitution cocktail I (**c**, **d**). Scale bars = 0.5 μm

The natural autolysis of the brain samples dismantled the ultrastructure and could not be reversed with the HFM. However, those structures that had not been degraded by autolysis, such as the myelin sheaths, appeared to be better preserved after HFM and freeze substitution with cocktail I. Moreover, this cocktail also resulted in consistently improved myelin sheath preservation with respect to standard embedding. Whereas the myelin sheaths remained visible even after long post-mortem times, finer structures, such as microtubules in the axons, were increasingly degraded with an increasing PMI. These drastic losses of ultrastructural details in the tissue were due to autolytic processes after the death of the patient. Other effects of autolysis were the enlargement of the coarse ER and loss of its ribosomes, swelling of the mitochondria and the fact that the chromatin in the nuclei became increasingly coarse (Fig. 4), resulting in an “empty” appearance of the cytoplasm. (i.e. it appeared void of compartments). After long post-mortem times, we were not able to recognize many of these compartments any more, instead, vacuole-like structures became apparent.

**Fig. 5:**
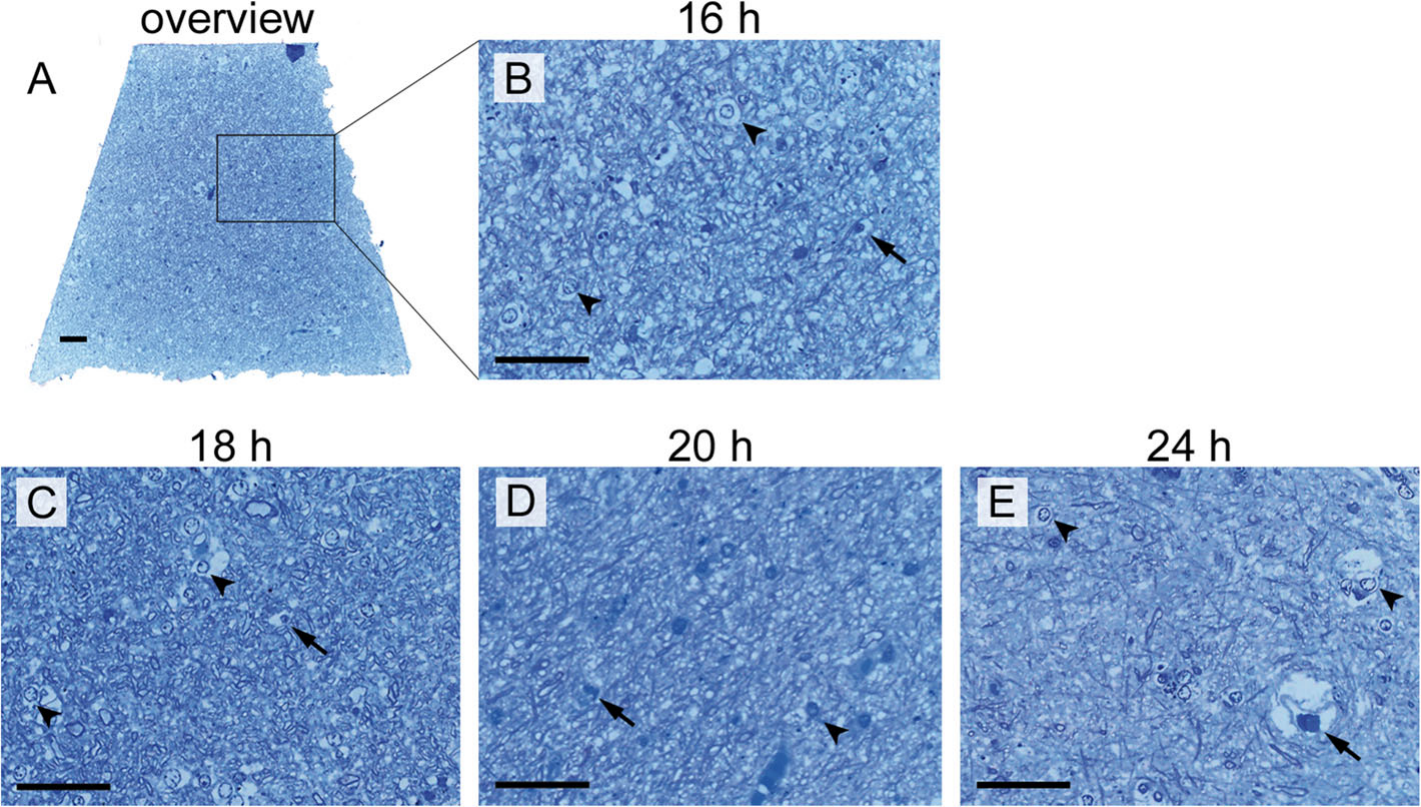
Semi-thin sections of human brain samples. All samples were prepared with cryo-substitution cocktail I, and after cutting ultra-thin slices 500 nm thick semi-thin slices were dyed with toluidine blue. The arrows indicate vessels with a light halo and the arrowheads glia cells with halo. **a** Case 1, PMI 16 h, frontal grey matter, zoom in is indicated with black square and shown in (**b**), **c** Case 2, PMI 18 h, frontal withe matter, **d** Case 3, PMI 20 h, frontal white matter, **e** Case 4, PMI 24 h, frontal withe matter. Scale bars = 20 μm

**Fig. 6:**
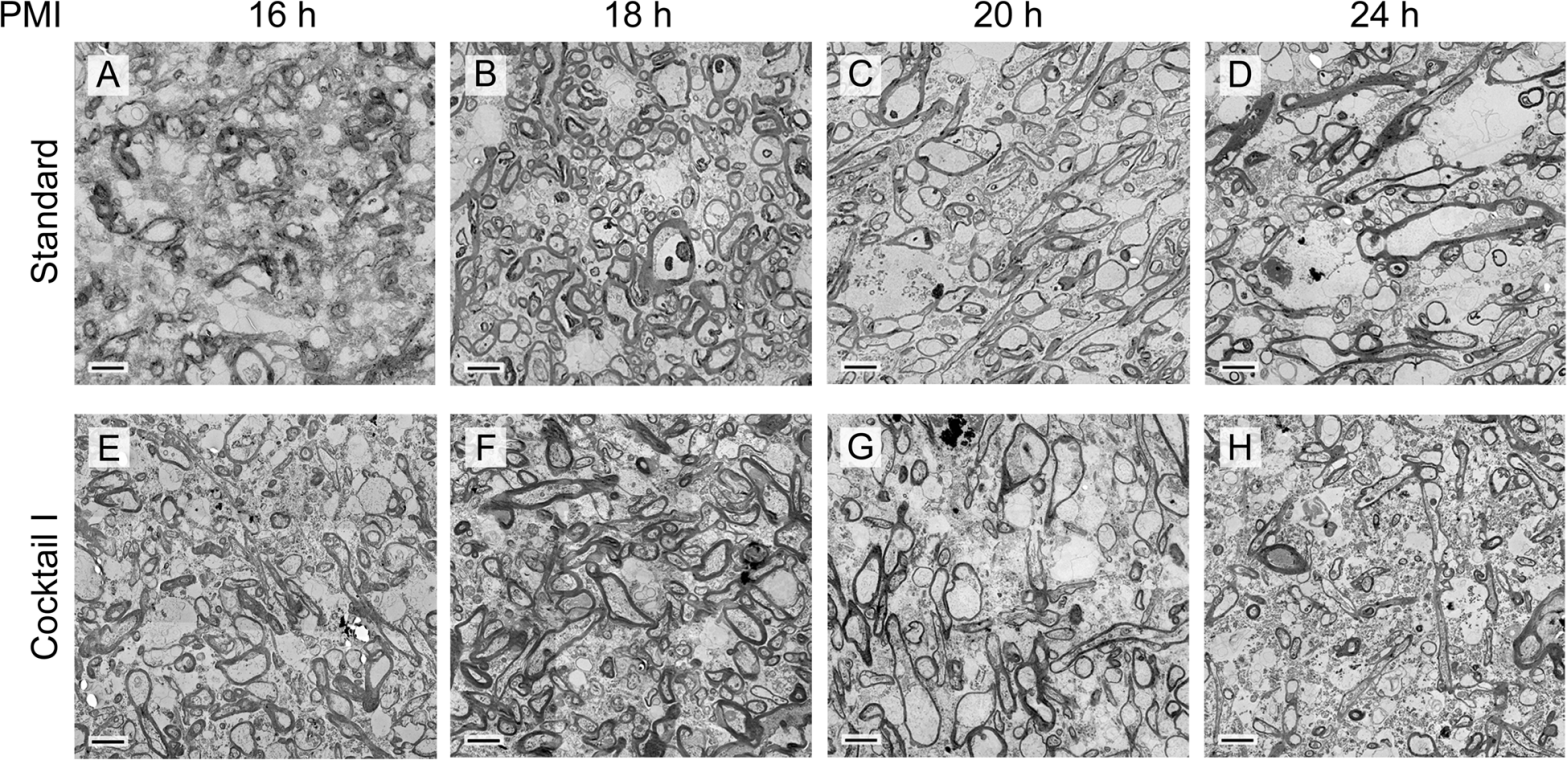
Comparison of human brain samples with different PMIs prepared with standard embedding and hybrid freezing. Overview micrographs (3 × 3 montages) at low magnification (1500x) (**a**-**d**) samples with increasing PMI (case 1–4) prepared with standard embedding method; **e**-**h** Samples of the same area from the same cases processed with cryo-substitution cocktail I, hybrid freezing. Scale bars = 1 μm

Light microscopic signs of autolysis were dilations around the vessels and the glia cells. These increased dramatically in size with increasing PMI (Fig. 5). The samples taken from case 1 (PMI 16 h, frontal grey matter) showed a dense organization of the neuronal tissue with a high number of finer structures. In the samples taken from frontal white matter (Fig. 5c-e), with increasing PMI, the organization of the tissue loosened more and more, and vacuole-like structures (putative degraded cellular compartments that could not be recognized any more) dominated instead (Fig. 5 and Fig. 6). Samples with a long PMI (case 4) showed an increased number of vacuole-like structures and hardly any structural details in the surrounding matrix.

Nevertheless, the samples processed with the HFM with cocktail I appeared to have a denser organization of the tissue and better preservation of finer details than those samples processed with standard embedding (compare Fig. 6a-d with Fig. 6e-h).

### Analysis of myelin sheath thickness (g-ratio) and density

**Fig. 7:**
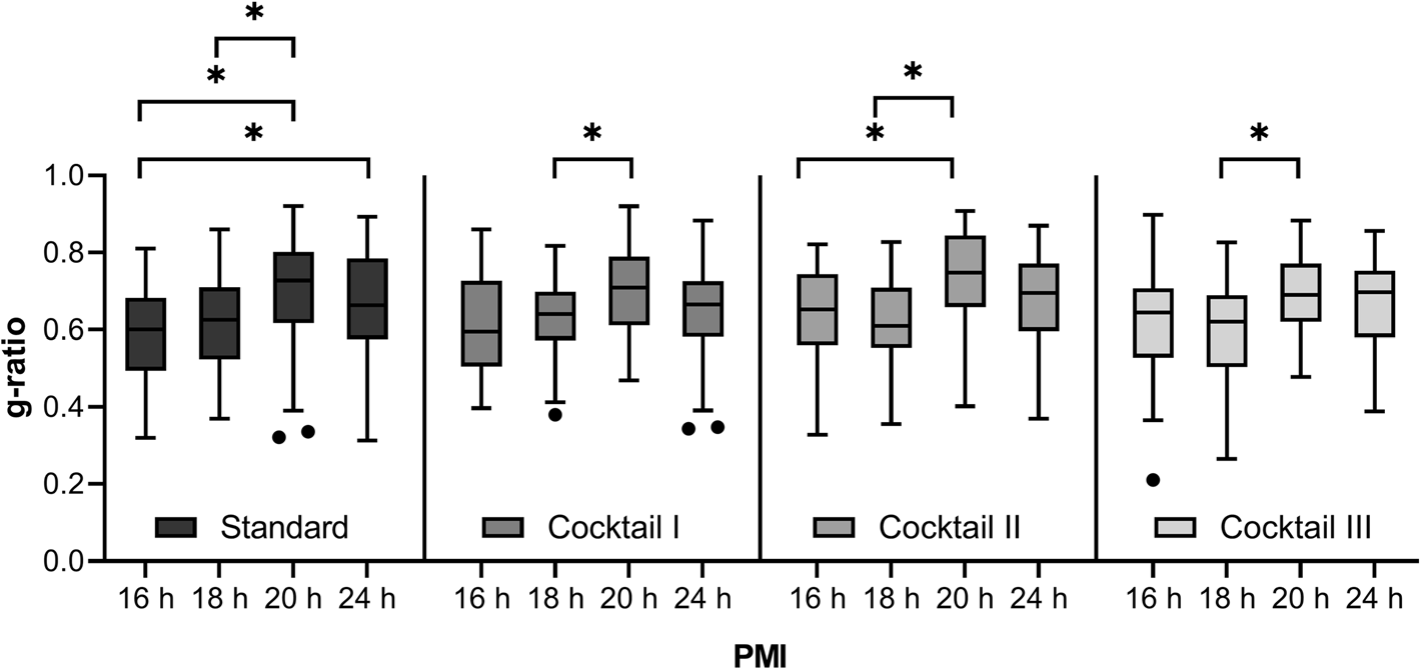
Analysis of g-ratios in human frontal lobe with different PMI’s and variable substitution cocktails compared to standard embedding. Boxplot with Tukey whiskers, ANOVA Scheffé post-hoc analysis revealed a significant difference *(*p* < 0.05) between seven samples

G-ratios were measured for each sample showing that each individual had their own g-ratio for each sample. In some cases, the individual g-ratios differed significantly from each other. For example, the g-ratios of case 2 and case 3 showed a significant difference between all four ways of processing the tissue, Fig. 7. With standard resin embedding, the mean values ± standard deviation of the g-ratios were as follows: case 1: (PMI 16 h) 0.59 ± 0.12, case 2: (PMI 18 h) 0.62 ± 0.12, case 3: (PMI 20 h) 0.70 ± 0.15, and case 4 (PMI 24 h) 0.67 ± 0.12. With cryo-substitution cocktail I, case 1: 0.61 ± 0.12, case 2: 0.64 ± 0.10, case 3: 0.70 ± 0.11 and case 4: 0.65 ± 0.12. For cocktail II the g-ratios were for case 1: 0.65 ± 0.12, case 2: 0.62 ± 0.12, case 3: 0.73 ± 0.12, case 4: 0.68 ± 0.12. After applying cocktail III, the ratios were 0.63 ± 0.14 for case 1, 0.61 ± 0.13 for case 2, 0.69 ± 0.10 for case 3, and 0.67 ± 0.12 for case 4. There was no statistically significant difference between g-ratios when the different substitution cocktails are compared in the same case as determined by one-way ANOVA, case 1: (*F* (3,232) = 1.957, *p* = 0.121), case 2: (*F* (3,213) = 0.515, *p* = 0.673), case 3: (*F* (3,231) = 0.448, *p* = 0.719) and case 4: (*F* (3,226) = 0.418, *p* = 0.740).

**Fig. 8:**
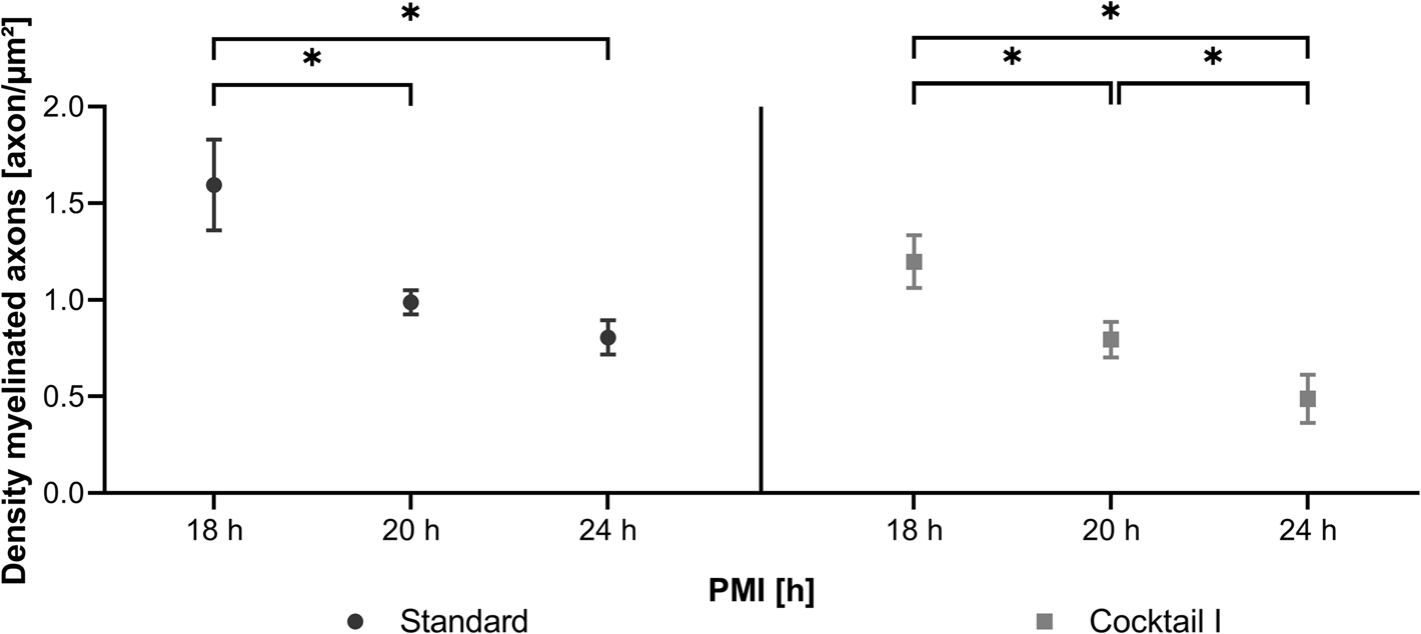
Analysis of the density of myelinated axons in human frontal lobe with different PMI’s prepared with standard embedding and cryo-substitution cocktail I. Mean and standard deviation values of the number of myelinated axons per square micrometer image are shown. One-way ANOVA with Scheffé post-hoc analysis revealed a significant difference *(*p* < 0.05) between the standard embedding and cryo-substitution cocktail I

A comparison of the densities of myelinated axona in sections of human frontal white matter revealed two facts. Firstly, these densities decreased with longer PMI, as can be seen in Fig. 8. Second, the samples prepared with the standard embedding approach showed significantly higher densities of myelinated axons than hybrid frozen samples (Fig. 8). In total, over 6000 myelinated axons were counted in ~ 6000 μm^2^ image area. A one-way ANOVA revealed significant differences between the two embedding methods regarding the density of the myelinated axons, standard embedding: (*F* (33,212), *p* = 0.000), cocktail I: (*F* (22,943), *p* = 0,000). With standard resin embedding, the mean values ± standard deviation of the axon density were as follows: case 2 (PMI 18 h): 1.59 ± 0.22, case 3 (PMI 20 h): 0.99 ± 0.06, case 4 (PMI 24 h): 0.80 ± 0.08. After preparation with cryo-substitution cocktail I, the axon density was (PMI 18 h): 1.20 ± 0.12 for case 2, (PMI 20 h): 0.80 ± 0.08 for case 3, and (PMI 24 h): 0.49 ± 0.11 for case 4.

## Discussion

A good preservation of the ultrastructure with a high contrast is essential for any electron microscopic analysis. It is important to preserve the subcellular structures so that they can be recognized and analyzed in detail. This is of particular importance if the samples are intended for further analyses like EM immuno-labeling [11, 24] or analytical electron microscopy [40]. High pressure freezing followed by freeze substitution has the advantage that the almost native state of the tissue can be preserved. Due to the lack of fixation, there are no fixation artifacts and because of the low temperatures, there is less shrinking during dehydration. Challenging is that for high pressure freezing, the tissue must be cut at around 200 μm thickness. This is extremely difficult to achieve with brain tissue as fresh brain tissue is soft due to its high water content. Moreover, the tissue must ideally be frozen within seconds after dissection. Chemical fixation has the advantage of a larger possible sample size. Moreover, the chemical fixative can be carried into the laboratory where the tissue is dissected. So there is no time delay between the start of the fixing process and the dissection. Here we applied a new Hybrid freezing method (HFM) that combines the advantages of standard chemical fixation with the advantages of HPF and cryo-substitution. Immediately after dissection, the tissue was chemically fixed in aldehyde solution, post fixed in osmium tetroxide, cut at 150 μm thickness, high pressure frozen, and freeze substituted. We showed that using HFM we could obtain better contrast and better myelin sheath preservation with respect to chemical fixation and dehydration at room temperature (standard method). One previous study of human brain tissue has already described a combination of aldehyde fixation, HPF and cryo-substitution [32], but our study has significantly expanded this previous findings by establishing a post-fixing step in osmium tetroxide prior to high pressure freezing and by systematically validating several different substitution cocktails. We showed that osmium tetroxide post fixation improves the preservation of the myelin sheaths and that a freeze substitution cocktail containing both osmium tetroxide and uranyl acetate leads to an enhanced contrast and more reliable results as compared to other cocktails. We are thus able to recommend a standard protocol that includes post-fixation with osmium tetroxide and a substitution cocktail containing both osmium tetroxide and uranyl acetate. Compared to the protocol described by Sosinsky et al. [32], it was also possible to significantly reduce the substitution time by continuous agitation [9] during the substitution. Besides better myelin sheath preservation, the osmium step before the HPF might reduce the risk of infections, which is an important point when working with human brain samples. It was shown that some cells of *Mycobacterium tuberculosis* were still viable after application of 2% glutaraldehyde overnight [21]. The high water content in the brain tissue [31], compared to other tissue, not only complicates the production of fresh samples suitable for HPF, but also constitutes a challenge especially when HPF is used, because the sample must be frozen before the cell water can turn into ice crystals [4, 22]. Therefore, hexadecane was not only used as a cryoprotectant in this study [20], but also as a means to achieve better pressure and temperature transfer in HPF [3], which proved successful. The dehydration of the samples in RT is known to cause damage to the ultrastructure, in particular the shrinkage and the extraction of some cellular components [24]. Dehydration in freeze substitution is less harmful due to a partial preservation of the hydration shell of the proteins [24] at the low substitution temperatures. In this study, cocktail I, a combination of osmium tetroxide and uranyl acetate dissolved in acetone, showed the highest contrast compared to the other substitution cocktails (see Fig. 1). Uranyl acetate is known to enhance contrast. According to Weibull et al. [36], it also stabilizes lipids against extraction. Curiously, the best and most reliable results were achieved by adding 2% osmium tetroxide to the substitution cocktail, even though the tissue samples had already been post-fixed with osmium tetroxide. The extra osmium tetroxide apparently helped to preserve the fine structural arrangement of the myelin sheaths. Other authors often add either water [3, 34] or tannic acid [15, 26, 32] to their substitution cocktails. Both procedures increased the contrast or structural preservation, but did not improve the results in our hands, as was shown in initial experiments (data not shown). While hybrid freezing helped preserve the myelin sheaths, we could show that the PMI remains a key factor regarding the quality of the ultrastructural preservation, which is in agreement with Glausier et al. [7], and is especially important for human brain biopsy samples [17]. The longer the PMI, the more signs of autolysis were visible, such as an increase in vacuoles that are void of structural material, as described by Xu et al. [37]. Moreover, the axon densities decreased significantly with decreasing PMI. Perpetuating this trend towards lower axon densities with higher PMIs, brain samples with an even longer PMI (36 h) displayed an even stronger decrease in myelinated axonal density down to only 0.14–0.19 axons / μm^2^, [18]. To avoid the effect of autolysis, some authors [5, 30] even recommend not to use samples with a PMI longer than 7 or 8 h, which would be difficult to achieve both for practical and for ethical reasons. The fact that each sample had a significantly lower axon density after HFM than after chemical fixation may seem counterproductive at first glance. But tissue has been shown to shrink during dehydration at room temperature (up to 2%, [14]). Hence the lower axon density we determined in the hybrid frozen samples may be closer to the native state of the tissue than the higher densities we determined after classical embedding. When comparing the g-ratios of the samples processed with the different approaches, no significant influence on g-ratios can be seen. However, regardless of the embedding method, some g-ratio values between individuals differed significantly from each other, which shows that the differences between individuals appear to be greater than the variations due to different methods processing the tissue. Those significant differences found may be due either to individual differences or to differences in PMI. These findings are in accordance with Liu and Schumann [18], who reported no significant difference of g-ratios between formalin-fixed and flash-frozen human brain samples.

## Conclusion

We established and validated a novel hybrid freezing EM preparation method for human brain samples. While we were not able to counteract autolytic processes, we were still able to visualize many fine structural features despite long post mortem intervals. We recommend a protocol in which the ultrastructure of human brain tissue can be preserved despite long PMIs. This protocol will make it easier to perform further analyses with the electron microscope than with standard protocols.

## Data Availability

Not applicable

## Abbreviations

CCD: Charge-coupled device
EM: Electron microscopy
ER: Endoplasmic reticulum
FS: Freeze substitution
FWF: Austrian Science Fund
HFM: Hybrid freezing method
HPF: High pressure freezing
PMI: Post-mortem interval
ROI: Region of interest
RT: Room temperature
